# Complement anaphylatoxin C4a inhibits C5a-induced neointima formation following arterial injury

**DOI:** 10.3892/mmr.2014.2176

**Published:** 2014-04-24

**Authors:** YAN ZHAO, HENG XU, WENHUI YU, BAO-DONG XIE

**Affiliations:** 1 Department of Emergency, Second Affiliated Hospital of Harbin Medical University, Harbin, Heilongjiang 150086, P.R. China; 2 Department of Vascular Surgery, Heilongjiang Provincial Hospital, Harbin, Heilongjiang 150001, P.R. China; 3 Department of Peripheral Vascular Surgery, The First Affiliated Hospital of Heilongjiang University of Traditional Chinese Medicine, Harbin, Heilongjiang 150001, P.R. China; 4 Department of Cardiovascular Surgery, Second Affiliated Hospital of Harbin Medical University, Harbin, Heilongjiang 150086, P.R. China

**Keywords:** atherosclerosis, neointima formation, complement anaphylatoxin C5a, complement anaphylatoxin C4a, macrophage

## Abstract

Interactions between complement anaphylatoxins have been investigated in numerous fields; however, their functions during arterial remodeling following injury have not been studied. The inhibitory effect of complement anaphylatoxin C4a on neointima formation induced by C5a following arterial injury was investigated. Mice were subjected to wire-induced endothelial denudation of the femoral artery and treated with C5a alone or C5a + C4a for two weeks. C4a significantly inhibited C5a-induced neointima formation and the expression of CD68, F4/80, tumor necrosis factor-α (TNF-α), interleukin-6 (IL-6) and monocyte chemotactic protein-1 (MCP-1). *In vitro*, although C4a did not directly inhibit the migration, proliferation or the expression of vascular cell adhesion molecule-1 (VCAM-1) of C5a-induced vascular smooth muscle cells (VSMCs), C5a-pretreated conditioned medium-induced migration, proliferation and VCAM-1 expression of VSMCs were suppressed when VSMCs were exposed to conditioned medium from C4a-pretreated macrophages. In addition, C5a-induced TNF-α, IL-6 and MCP-1 expression, Ca^2+^ influx and extracellular signal-regulated kinase (ERK) activation in macrophages were suppressed by C4a. C4a inhibits C5a-induced neointima formation, not by acting directly on VSMCs, but via a macrophage-mediated reaction by inhibiting the Ca^2+^-dependent ERK pathway in macrophages.

## Introduction

Atherosclerosis is an inflammatory disease driven by the interaction of inflammatory cells and the vascular wall (1,2). Vascular neointimal hyperplasia is a key event in arteriosclerosis and several other vascular diseases (3,4). Neointimal hyperplasia is an abnormal increase in the cell population within the innermost layer of an arterial wall (5). The underlying causes of neointimal hyperplasia include the migration, proliferation and apoptosis of vascular smooth muscle cells (VSMCs) provoked by endovascular injury or perivascular injury (6,7). According to the mechanisms above, the neointima consists mainly of VSMCs and the migration and proliferation of VSMCs are critical to neointimal hyperplasia following arterial injury (8). Complement components have been identified in neointimal plaques and atherosclerotic tissues (9,10). Complement anaphylatoxin C5a is a risk factor for atherosclerosis progression. Following arterial injury, C5a leads to pro-inflammatory activation and neointima formation during arterial remodeling (11). C5a affects the migration and proliferation of VSMCs through different pathways. C5a is able to bind to the C5a receptor on VSMCs and induce the autocrine signaling of factors, including vascular cell adhesion molecule-1 (VCAM-1) and platelet derived growth factor (PDGF) (11). It also binds to the receptor on macrophages and provokes the production of large amounts of cytokines and chemokines, including tumor necrosis factor-α (TNF-α), interleukin-6 (IL-6) and monocyte chemotactic protein-1 (MCP-1) (12), that accelerate neointimal hyperplasia by promoting the migration and proliferation of VSMCs (13).

C4a and C5a are liberated from the N-terminal region of the parental protein α-chain. Although they are similar in terms of molecular structure, it has been demonstrated that C5a is able to act on numerous types of cells; however, C4a is restricted to monocytes/macrophages in phagocytic leukocytes (14). The interactions between C5a and other complement anaphylatoxins have been investigated in numerous fields; however, the interactive role of C4a and C5a during arterial remodeling following injury has not been studied. Since C5a is able to promote the migration and proliferation of VSMCs via a macrophage-mediated inflammatory reaction, and C4a is able to inhibit the chemoattractant and secretagogue induced by C5a in monocytes/macrophages (15), the present study sought to determine if C4a inhibits C5a-induced neointima formation following arterial injury in the complement cascades.

In the present study, to the best of our knowledge, the hypothesis that C4a inhibits C5a-induced neointima formation following arterial injury was tested for the first time, and the results indicated that this may be a novel therapeutic strategy for neointimal hyperplasia.

## Materials and methods

### Animals

Animal care and protocol for this study were in accordance with the Animal Experiment Guidelines of Harbin Medical University (Harbin, China) and ethical approval was obtained from Harbin Medical University. C57BL/6N, 16-week-old mice (Fuzhong Biotechnology, Shanghai, China) were subjected to transluminal endovascular wire injury of the bilateral femoral artery, as reported previously (8). All the mice were fed normal chow and water *ad libitum* and kept under normal diurnal cycle at room temperature (22°C). Over a course of two weeks, recombinant C5a (Sigma, Shanghai, China; 1 μg/25 g of body weight) and recombinant C4a (1 μg/25 g of body weight) were administered via an Alzet mini-osmotic pump (American Health & Medical Supply International Corp, Chengdu, Sichuan, China) into the subcutaneous perifemoral artery following endovascular injury, according to a previously described method (16). Image analysis software was used to measure the neointima and media of the femoral artery. The relative mRNA expression levels of CD68 and F4/80 in femoral arterial tissue were assessed by quantitative polymerase chain reaction (qPCR), and the protein levels of TNF-α, IL-6 and MCP-1 in femoral arterial tissue were assessed by western blot analysis.

### Preparation of recombinant C4a

C4a cDNA was prepared by PCR using total mRNA derived from HepG2 cells. The confirmed nucleotide sequence of the PCR product was subcloned into the expression vector pET32a flanked by *Bam*HI and *Eco*RI restriction sites, and each plasmid cDNA was transformed to DH5α competent cells. Rosetta-gami B (DE3) Lys-S cells transformed with each expression vector were cultured in Lennox Broth medium containing ampicillin, chloramphenicol, kanamycin and tetracycline until the 600 nm absorbance of the bacterial suspension reached 0.6. Each suspension was mixed with 1 mM of isopropyl 1-thio-β-d-galactoside and incubated for an additional 4 h. Following centrifugation, the cultured E. coli cells were resuspended into 1/10 culture volume (50 ml) of 20 mM Tris-HCl containing 200 mM NaCl and 10 mM EDTA (pH 8.0). The bacteria were lysed by sonication in the presence of 1% Triton X-100. Following centrifugation, the extracted recombinant proteins were separated using a Hi-Trap™ Chelating HP column preloaded with 100 mM NiSO_4_ (GE Healthcare Life Sciences, Piscataway, NJ, USA). The purity of Trx-His-S-tag recombinant C4a was checked using SDS-PAGE. The recombinant C4a was dialyzed in phosphate-buffered saline (PBS) containing 1 mg/ml of bovine serum albumin (BSA; Sigma) and stored at −70°C until use.

### Cells

#### Primary culture of VSMCs

After the C57BL/6N mice were sacrificed, the aortas were separated and transferred into 10 cm dishes containing Dulbecco’s modified Eagle’s medium (DMEM). The endothelium was injured by prodding three to five times with a sterilized cotton swab. Endothelium-injured aortas were placed down and then cut into 0.2×0.2 cm squares, which were carefully transferred into six-well multiplates, two to three squares per well, containing DMEM + 20% fetal calf serum (FCS) culture medium (Sigma). Three to five generations of VSMCs were used in this experiment. VSMCs were exposed to C5a (10^−8^ M) in the presence or absence of C4a (10^−8^ M) or exposed to the conditioned medium of macrophages that had been pretreated with C5a in the presence or absence of C4a, following which the migration and proliferation of VSMCs were investigated and VCAM-1 expression was assessed by flow cytometry.

#### Primary culture of peritoneal macrophages

C57BL/6N mice were injected intraperitoneally with 4.0 ml of 4% thioglycollate (Difco Laboratories, Franklin Lakes, NJ, USA) and peritoneal macrophages were isolated from peritoneal lavage liquid three days later. Peritoneal macrophages were cultured in RPMI-1640 medium supplemented with 10% FCS for three days. Subsequently, C5a (10^−8^ M) was added to untreated macrophages or those that had been pretreated with C4a (10^−8^ M) for 10 min. Then, TNF-α, IL-6 and MCP-1 expression, Ca^2+^ influx and ERK activation in macrophages were assayed, respectively, by ELISA, a calcium imaging system and western blot analysis.

### Quantification of neointimal hyperplasia

The mice were sacrificed and the femoral arteries were harvested two weeks after surgical intervention. Arterial tissue was fixed in 10% formalin and embedded in paraffin. The middle segment of the artery was cut into five serial cross-sections at 200 μm intervals. Following elastica van Gieson staining for connective tissue, areas of the neointima and artery were measured using image analysis software (Image J; http://imagej.nih.gov/ij/), as previously described (17).

### qPCR

Total RNA was extracted from femoral arterial tissue and relative mRNA was normalized to 18s. The following primers were used: F4/80 forward, 5′-GAGATTGTGGAAGCATCCGAGAC-3′ and reverse, 5′-GATGACTGTACCCACATGGCTGA-3′; Cd68 forward, 5′-CATCAGAGCCCGAGTACAGTCTACC-3′ and reverse, 5′-AATTCTGCGCCATGAATGTCC-3′; 18s forward, 5′-GTAACCCGTTGAACCCCATT-3′ and reverse, 5′-CCATCCAATCGGTAGTAGCG-3′. qPCR was performed using the ABI 7300 Fast real-time PCR system (Applied Biosystems, Foster City, CA, USA).

### Western blot analysis

Electrophoresis was performed using a vertical slab gel with a 12% polyacrylamide content according to the method by Laemmli (18). The transfer of proteins from the SDS polyacrylamide gel to a membrane was performed electrophoretically according to the method by Kyhse-Andersen (19) with certain modifications using a Semi Dry Electroblotter (Sartorius AG, Goettingen, Germany) for 90 min with an electric current of 15 V. The membrane was treated with Block Ace™ (4%) for 30 min at 22°C. The first reaction was performed using rabbit immunoglobulin (IG) G antibodies against TNF-α, IL-6, MCP-1 and unmodified protein or against phosphorylated protein of ERK 1/2 (100 ng/ml; Sigma) in PBS containing 0.03% Tween-20 for 1 h at 22°C. Following washing in the same buffer, the second reaction was performed using horseradish peroxidase (HRP)-conjugated anti-rabbit goat IgG (20 ng/ml) for 30 min at 22°C. Following washing, the enhanced chemiluminescence (ECL) reaction was performed on the membrane using the ECL Plus Western Blotting Detection System™ (GE Healthcare Life Sciences).

### Flow cytometry

VSMCs (2×10^6^ cells/ml) were incubated for 24 h with C5a (10^−8^ M) in the presence or absence of C4a (10^−8^ M) or with conditioned medium of macrophages that had been pretreated with C5a in the presence or absence of C4a. VSMCs were stained with anti-mouse CD106 (VCAM-1)-phycoerythrin rat IgG2a (Sigma) antibodies as the isotype control for 30 min on ice. The cells were analyzed using a FACS Calibur flow cytometer (BD Biosciences, Tokyo, Japan).

### Migration assay

The migration assay was performed using a 48-well chamber migration assay kit with Nuclepore filters (Nuclepore, Pleasanton, CA, USA) with a pore size of 8 μm according to the method by Falk *et al* (20). For preparation, the upper wells were coated with 0.01% collagen for 30 min of incubation at 37°C. Next, VSMCs (5×10^4^ cells/well) were seeded into the top wells. The chemotactic medium was added to the lower wells. Following being incubated at 37°C for 8 h, the cells that had migrated to a lower filter surface were fixed with 4% paraformaldehyde in PBS for 10 min at room temperature and stained with hematoxylin and eosin Y. Cell migration was defined as the number of cells that had migrated to a lower filter surface.

### Proliferation assay

The proliferation assay was performed by using the Cell Counting kit-8 (CCK-8; Dojindo Molecular Technologies, Inc., Kumamoto, Japan) according to the manufacturer’s instructions. Firstly, a suspension of VSMCs (5,000 cells/100 μl/well) was loaded into the wells of a 96-well plate. The cells were then incubated at 37°C for 24 h. CCK-8 solution (10 μl) was added to each well and the cells were incubated for 3 h at 37°C. A microplate reader was then used to measure the absorbance at 450 nm. According to the prepared standard curve, the relative cell numbers were calculated.

### ELISA

C5a (10^−8^ M) was added to untreated macrophages or those that had been pretreated with C4a (10^−8^ M) for 10 min. Then, the supernatant was collected for analysis of TNF-α, IL-6 and MCP-1 by using the mouse ELISA kit (Sigma). The ELISA plates were coated with 100 μl capture antibody per well at 4°C overnight. Following an appropriate wash, 200 μl of assay dilution buffer was added per well for inhibition at room temperature for 1 h. The sample and serial dilutions of the standards were added to the plate and incubated at 4°C overnight. Following coating with detection antibody, avidin-HRP was added and incubated at room temperature for 30 min. The substrate 3,3′,5,5′-tetramethylbenzidine was added and incubated for 15 min. Finally, 2N H_2_SO_4_ was added to terminate the reaction and the absorbance at 450 nm was measured using an ELISA reader (MTP-800 Microplate reader; Corona Electric, Tokyo, Japan).

### Measurement of cytoplasmic Ca^2+^ influx

Ca^2+^ imaging was performed as described previously (21). The macrophages (2×10^6^ cells/ml) were loaded with calcium-sensitive Fura 2-AM (1 μM) in Ca^2+^-free buffer (Hanks’ balanced salt solution containing 20 mM of 4-(2-hydroxyethyl)-1-piperazineethanesulfonic acid and 1% BSA; pH 7.4) for 30 min at 37°C according to the manufacturer’s instructions (Dojindo Laboratories, Inc.). The samples were placed directly into the cell suspension following a 3 min baseline recording. The recordings were made with an F-2500 calcium imaging system from FL Solutions (Hitachi, Tokyo, Japan) that calculated the ratio of fluorescent signals obtained at 37°C with excitation wavelengths at 340 and 380 nm, and with an emission wavelength at 510 nm. The excitation wavelengths at 380 nm and 340 nm were used to measure the free Fura-2 and the Ca^2+^-bound Fura-2, respectively. The fluorescent activities of 340 nm/500 nm (F1) and of 380 nm/500 nm (F2) and the ratio (R) of F1 to F2 were recorded by the spectrophotometer at the indicated time. The Ca^2+^ concentration (C) was then calculated using the following formula: C = 224 × R, where 224 is the K_d_ number.

### Statistical analysis

Data are expressed as the mean ± standard error or mean ± standard deviation. Each experiment was repeated at least three times. The Student’s t-test was used and P<0.05 was considered to indicate a statistically significant difference.

## Results

### Inhibitory effect of C4a on C5a-induced neointima formation

**Figure 1:**
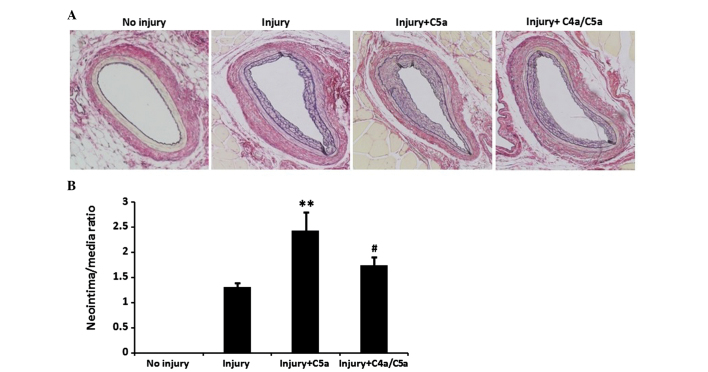
Inhibitory effect of C4a on C5a-induced neointima formation. Mice were treated with C5a (1 μg/25 g of body weight) or C5a + C4a (1 μg/25 g of body weight) by mini-osmotic pumps for two weeks following injury. Following treatment with C5a, the neointimal area increased significantly. The increased neointimal area was significantly reduced following C4a treatment. (A) Representative image of an elastic Van Gieson-stained neointima from femoral artery samples harvested two weeks after endovascular injury (original magnification, ×1). (B) Quantification of A. Data are expressed as the mean ± standard error (n=5). P<0.05 was considered to indicate a statistically significant difference (^**^P<0.01, injury + C5a vs injury; ^#^P<0.05, injury + C5a/C4a vs injury + C5a).

**Figure 2:**
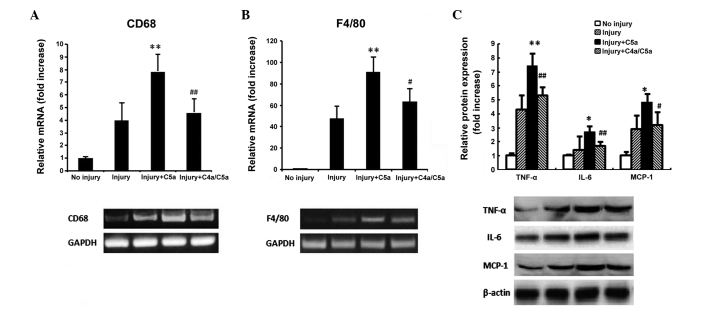
Inhibitory effect of C4a on C5a-induced mRNA expression of CD68 and F4/80 and the protein levels of TNF-α, IL-6 and MCP-1 in femoral artery tissue. Mice were treated with C5a (1 μg/25 g of body weight) or C5a + C4a (1 μg/25 g body weight) by mini-osmotic pumps for two weeks after injury. Following treatment with C5a, the mRNA expression of (A) CD68, (B) F4/80 as well as the protein levels of (C) TNF-α, IL-6 and MCP-1 increased significantly. The increased mRNA expression of (A) CD68, (B) F4/80 and protein levels of (C) TNF-α, IL-6 and MCP-1 were significantly reduced following C4a treatment. Data are expressed as the mean ± standard error (n=5). P<0.05 was considered to indicate a statistically significant difference. (^**^P<0.01, ^*^P<0.05, injury + C5a vs injury; ^##^P<0.01, ^#^P<0.05, injury + C5a/C4a vs injury + C5a). TNF-α, tumor necrosis factor-α; interleukin-6; MCP-1, monocyte chemotactic protein-1.

To investigate the inhibitory effect of C4a on C5a-induced neointima formation, the mice were treated with C5a (1 μg/25 g of body weight) or C5a + C4a (1 μg/25 g of body weight) by mini-osmotic pumps for two weeks after injury. Following treatment with C5a, the neointimal area increased significantly (Fig. 1; P<0.01). C5a also increased the mRNA expression of CD68 and F4/80 (Fig. 2A and B; P<0.01) and the protein levels of TNF-α, IL-6 and MCP-1 (Fig. 2C; P<0.01) in femoral artery tissue. However, following C5a and C4a treatment, the neointimal area was significantly reduced (Fig. 1; P<0.05). C4a also inhibited the mRNA expression of CD68 and F4/80 (Fig. 2A and B; P<0.05) and the protein levels of TNF-α, IL-6 and MCP-1 (Fig. c; P<0.05) in femoral artery tissue induced by C5a stimulation.

### Lack of inhibitory effect of C4a on VSMCs in response to C5a

**Figure 3:**
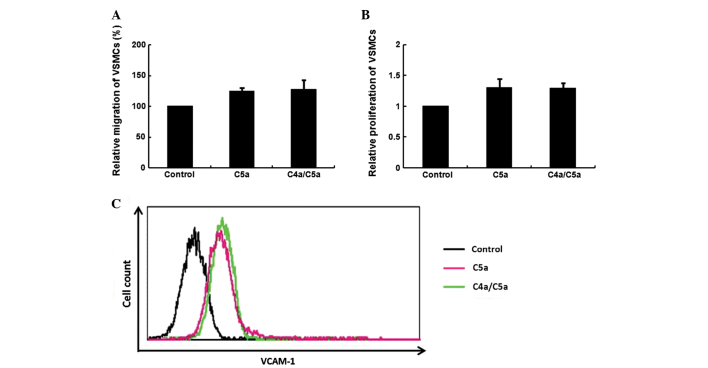
Lack of inhibitory effect of C4a on VSMCs in response to C5a. VSMCs were treated with C5a (10^−8^ M) in the presence or absence of C4a (10^−8^ M), then the migration, proliferation and VCAM-1 expression of VSMCs were investigated. (A) C4a did not inhibit C5a-induced low levels of migration, (B) proliferation or (C) VCAM-1 expression of VSMCs. Data were normalized to cell numbers and are expressed as the mean ± standard deviation (n=3). P<0.05 was considered to indicate a statistically significant difference. VSMCs, vascular smooth muscle cells; VCAM-1, vascular cell adhesion molecule-1.

Since VSMCs are critical in vascular remodeling following vascular injury (22), the effect of C4a on the VCAM-1 regulation of VSMCs was investigated. VSMCs were treated with C5a (10^−8^ M) in the presence or absence of C4a (10^−8^ M), then the migration, proliferation and VCAM-1 expression of VSMCs were investigated. C4a did not inhibit C5a-induced low levels of the migration (Fig. 3A), proliferation (Fig. 3B) or VCAM-1 expression of VSMCs (Fig. 3C).

### Inhibition of VSMCs by conditioned medium from C4a-pretreated macrophages

**Figure 4:**
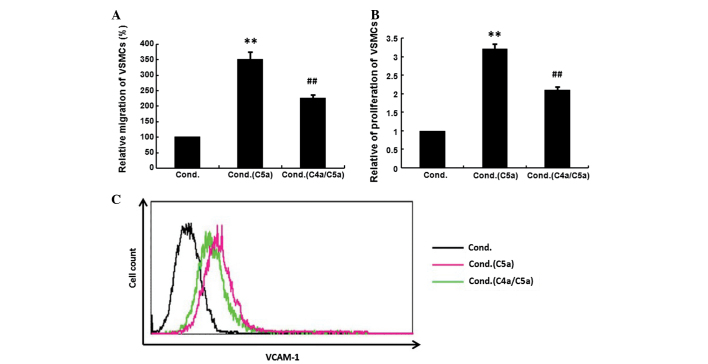
Inhibition of VSMCs by conditioned medium from C4a-pretreated macrophages. C5a (10^−8^ M) was added to untreated macrophages or those that had been pretreated with C4a (10^−8^ M), then the supernatant from macrophages was added to VSMCs. Conditioned medium from C5a-treated macrophages significantly increased the migration, proliferation and VCAM-1 expression of VSMCs (^**^P<0.01). The increased migration, proliferation and upregulation of VCAM-1 expression were significantly suppressed when VSMCs were exposed to the conditioned medium from C4a-pretreated macrophages (^##^P<0.01). Data were normalized with cell numbers and are expressed as the mean ± standard deviation (n=3). P<0.05 was considered to indicate a statistically significant difference. Cond, conditioned medium from single macrophages; Cond. (C5a), conditioned medium from C5a-treated macrophages; Cond. (C5a/C4a), conditioned medium from C5a/C4a-treated macrophages; VSMCs, vascular smooth muscle cells; VCAM-1, vascular cell adhesion molecule-1.

C5a (10^−8^ M) was added to untreated macrophages or those that had been pretreated with C4a (10^−8^ M), then the supernatant from macrophages was added to VSMCs. The migration, proliferation and VCAM-1 expression of VSMCs were investigated. The conditioned medium from C5a-treated macrophages significantly increased the migration, proliferation and VCAM-1 expression of VSMCs. The increased migration, proliferation and the upregulation of VCAM-1 expression were significantly suppressed when VSMCs were exposed to the conditioned medium from C4a-pretreated macrophages (Fig. 4; P<0.01).

### Inhibitory effect of C4a on macrophages in response to C5a

**Figure 5:**
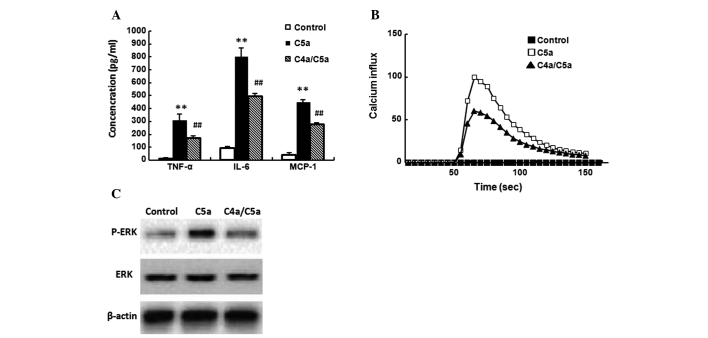
Inhibitory effect of C4a on macrophages in response to C5a. C5a (10^−8^ M) was added to untreated macrophages or those that had been pretreated with C4a (10^−8^ M) for 10 min. C5a significantly induced the expression of (A) TNF-α, IL-6 and MCP-1, (B) Ca^2+^ influx and (C) ERK activation in macrophages (^**^P<0.01). C4a significantly suppressed C5a-induced (A and B) TNF-α, IL-6 and MCP-1 expression and Ca^2+^ influx, and (C) inhibited C5a-induced ERK activation in macrophages (^##^P<0.01). Data were normalized to cell numbers and are expressed as the mean ± standard deviation (n=3). P<0.05 was considered to indicate a statistically significant difference. TNF-α, tumor necrosis factor-α; interleukin-6; MCP-1, monocyte chemotactic protein-1.

C5a (10^−8^ M) was added to untreated macrophages or those that had been pretreated with C4a (10^−8^ M) for 10 min. Then, TNF-α, IL-6 and MCP-1 expression, Ca^2+^ influx and ERK activation in macrophages were assayed, respectively. C5a significantly induced TNF-α, IL-6 and MCP-1 expression (Fig. 5A), Ca^2+^ influx (Fig. 5B) and ERK activation (Fig. 5C) in macrophages (P<0.01). C4a significantly suppressed C5a-induced TNF-α, IL-6 and MCP-1 expression and Ca^2+^ influx (Fig. 5A and B; P<0.01), and inhibited C5a-induced ERK activation (Fig. 5C). C4a alone did not induce the expression of TNF-α, IL-6 and MCP-1, Ca^2+^ influx or phosphorylation of ERK (data not shown).

## Discussion

The present study demonstrated, for the first time, to the best of our knowledge, that the inhibition of C5a by C4a limits neointima formation following arterial injury in wire-induced endothelial denudation of the femoral artery of mice. Although C4a and C5a are released from the N-terminal region of the parental protein α-chain and are similar in terms of molecular structure, they have different functions (23). C5a is a potent soluble anaphylotoxic and chemotactic inflammatory mediator (24). It is able to act on numerous types of cells by binding to the C5a receptor (25); as such, C5a induces pro-inflammatory activation and targets neointima formation following arterial injury (11). C4a was previously isolated from the inflammatory joint fluid of patients with rheumatoid arthritis and identified as the monocyte/macrophage migration inhibitory factor (26). The C4a receptor is restricted to monocytes/macrophages in phagocytic leukocytes (14). C4a is able to inhibit the chemoattractant and secretagogue induced by C5a in monocytes/macrophages (15). The present study investigated whether C4a was able to inhibit C5a-induced neointima formation via the complement cascade. The present study confirmed the stimulatory effect of C5a on neointima formation and demonstrated the protective effect of C4a treatment following injury.

VSMCs from the media are key in the progressive intimal thickening that leads to atherosclerosis and restenosis (22). Since VSMCs are critical in neointimal hyperplasia, the present study focused on those factors that are able to affect VSMCs. C5a is a risk factor for the progression of atherosclerosis (27) It affects the migration and proliferation of VSMCs through different pathways by a series of coordinated signals (11,28,29). C5a is able to bind to the C5a receptor on VSMCs (29) and induce the autocrine signaling of factors, including VCAM-1 and PDGF (11). VCAM-1 and PDGF are important in early atherogenesis, their expression is a marker of the transition of VSMCs to the synthetic phenotype within atherosclerotic lesions (30). Following arterial injury, inflammatory cells, including monocytes and macrophages, are recruited into neointimal sites (31). Consistent with that, our data demonstrated that the mRNA expression of CD68 and F4/80 (as indicators of macrophage accumulation) in femoral artery tissue increases significantly following treatment with C5a (Fig. 2A and B). C5a also binds to the receptors on inflammatory cells, particularly on macrophages, and provokes the production of large amounts of cytokines and chemokines, including TNF-α, IL-6 and MCP-1 (Fig. 2C) (12). TNF-α is a canonical inflammatory cytokine that promotes the migration and proliferation of VSMCs (13). IL-6 is a pleiotropic cytokine involved in pro-inflammatory and anti-inflammatory responses via regulating leukocyte function and apoptosis (32). MCP-1 is a potent chemoattractant that is essential in various inflammatory diseases involving the recruitment of monocytes/macrophages (33). These factors all accelerate neointimal hyperplasia by promoting the migration and proliferation of VSMCs (13,34).

With the receptor on VSMCs, C5a induced low levels of migration and proliferation as well as VCAM-1 expression in VSMCs; however, this migration, proliferation and VCAM-1 expression could not be suppressed by C4a. These results demonstrated that C4a did not directly act on VSMCs. There is no evidence verifying the presence of a VSMC C4a receptor and our data also suggest its absence in VSMCs. However, when C5a was added to untreated or C4a-pretreated macrophages and then the supernatant from macrophages was added to VSMCs, it was revealed that C5a-treated macrophages-conditioned medium, which contained cytokines TNF-α, IL-6 and chemokine MCP-1, significantly increased the migration and proliferation of VSMCs, and TNF-α further increased VCAM-1 expression in VSMCs. These results were consistent with previous studies (12,35). The increased migration, proliferation and upregulation of VCAM-1 expression were significantly suppressed when VSMCs were exposed to the conditioned medium from C4a-pretreated macrophages (Fig. 4). Regarding the underlying mechanism, the present study revealed that the C5a-induced expression of TNF-α, IL-6 and MCP-1 in macrophages was suppressed by C4a (Fig. 5A). These results confirmed the presence of a C4a receptor on macrophages.

**Figure 6:**
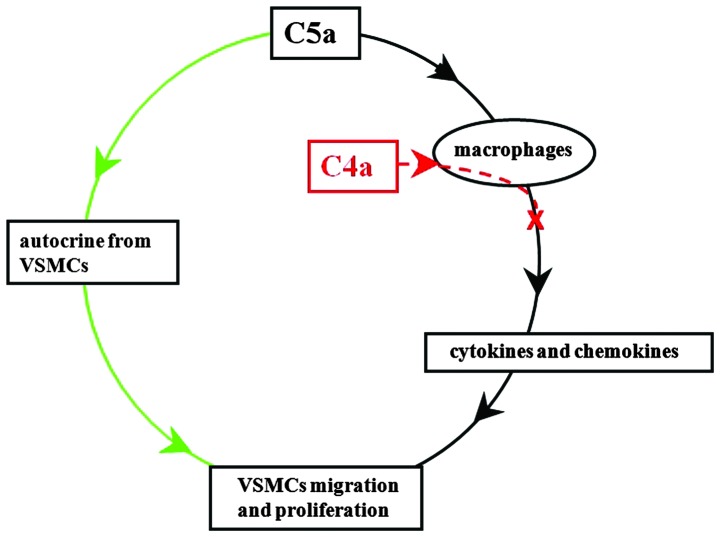
Inhibitory mechanism in neointima formation. The migration and proliferation of VSMCs are critical to neointimal hyperplasia following arterial injury. C5a can either act on VSMCs to induce the autocrine signaling of factors or act on macrophages to provoke the production of large amounts of cytokines and chemokines. C4a is not able to directly act on VSMCs; however, it is able to inhibit the migration and proliferation of VSMCs via a macrophage-mediated inflammatory reaction by inhibiting the production of cytokines and chemokines in macrophages. VSMCs, vascular smooth muscle cells.

It has been demonstrated that the release of cytokines and chemokines from C5a-stimulated macrophages is associated with cytoplasmic Ca^2+^ influx (36). C5a binds to the C5a-receptor and induces cytoplasmic Ca^2+^ influx (37), thereby stimulating the release of TNF-α, IL-6 and MCP-1 from macrophages (38). In order to better understand the protective mechanisms of C4a on C5a-induced neointima formation, the present study examined whether C4a was able to inhibit C5a-induced cytoplasmic Ca^2+^ influx in macrophages. C4a significantly suppressed cytoplasmic Ca^2+^ influx in macrophages (Fig. 5B). Furthermore, as the ERK1/2 pathway is a process that includes the authentic cytoplasmic Ca^2+^ influx (39), whether C4a was able to inhibit C5a-induced ERK activation in macrophages was investigated. It was observed that C4a effectively inhibited the ERK1/2 pathway, which included Ca^2+^ mobilization (Fig. 5C). These *in vitro* data are in accordance the *in vivo* findings of the present study. According to the mechanism described in Fig. 6, C4a inhibits C5a-induced neointima formation, not by acting directly on VSMCs, but via a macrophage-mediated inflammatory reaction by inhibiting the Ca^2+^-dependent ERK signaling pathway in macrophages. The complement cascade is a complex process, although the inhibition of C5a-induced neointima formation by C4a was demonstrated, the complex inhibitory process and the identification of a C4a-receptor requires further study.

The present study, to the best of our knowledge, is the first demonstration that treatment with C4a significantly reduces C5a-induced neointima formation following arterial injury, via a macrophage-mediated inflammatory reaction by inhibiting the Ca^2+^-dependent ERK signaling pathway in macrophages. The crucial role of C5a/C4a reactions in neointima formation following vascular injury is expected to provide important information for the development of novel clinical treatments for vascular diseases.
